# Complicated norovirus infection and assessment of severity by a modified Vesikari disease score system in hospitalized children

**DOI:** 10.1186/s12887-016-0699-2

**Published:** 2016-10-05

**Authors:** Pei-Lin Wang, Shih-Yen Chen, Chi-Neu Tsai, Hsun-Ching Chao, Ming-Wei Lai, Yi-Jung Chang, Chyi-Liang Chen, Cheng-Hsun Chiu

**Affiliations:** 1 Department of Pediatrics, Division of Pediatric Gastroenterology, Chang Gung Children’s Hospital, Chang Gung University College of Medicine, Taoyuan, Taiwan; 2 Division of Pediatric Gastroenterology, Department of Pediatrics, Chang Gung Children’s Hospital, Graduate Institute of Clinical Medical Sciences, Chang Gung University College of Medicine, Taoyuan, Taiwan; 3 Molecular Infectious Disease Research Center, Chang Gung Memorial Hospital, Taoyuan, Taiwan; 4 Department of Pediatrics, Division of Pediatric Infectious Diseases, Chang Gung Children’s Hospital, Chang Gung University College of Medicine, Taoyuan, Taiwan

**Keywords:** Gastroenteritis, Norovirus, Complications, Disease severity score

## Abstract

**Background:**

Norovirus (NoV) GII.4 is the most common genotype for norovirus gastroenteritis worldwide. New variants or subgenotypes are continuously emerging, thus posing a serious threat to child health.

**Methods:**

We compared retrospectively the clinical manifestations and complications of norovirus gastroenteritis in children from April, 2004 through December, 2012. NoV variants were analyzed to investigate the association of circulating viral strains with the complications. A modified disease severity score system based on Vesikari score system was devised and to evaluate disease severity.

**Results:**

Compared to the outbreak in 2004/2005 winter, significant higher incidence of complications in the later periods are: convulsive disorder (*p* < 0.001) in 2006/2007 winter gastrointestinal hemorrhage (*p* = 0.047) and severe abdominal pain or irritability (*p* = 0.033) in 2008/09/10 winter; gastrointestinal hemorrhage (*p* = 0.030), severe abdominal pain or irritability (*p* = 0.014), and prominent hyperthermia (fever >39 °C, *p* = 0.001) in 2011/2012 winter. GII.4 Den_Haag_2006b, GII.4 2010, GII.4 Sydney 2012, and GII.4 2012b were the predominant strains in the outbreaks after 2006. By the modified severity score system, severe norovirus disease occurred in 28.5 %, 32 %, 33.3 %, and 30.2 % of the patients in the four periods. A longer duration of hospitalization (*p* = 0.02) were found in those with high score irrespective of the year of admission.

**Conclusions:**

Our study demonstrated NoV outbreaks in northern Taiwan caused by different GII.4 variants that were associated with specific complications and uncommon clinical presentations. A modified severity score system first proposed in this study was able to identify severe cases with a longer hospital stay in NoV-infected children.

## Background

Acute gastroenteritis (AGE) is one of the most common infectious diseases and still a major cause of pediatric morbidity and mortality worldwide. The two most important viral agents in children are rotaviruses and noroviruses [1]. Norovirus (NoV) is genetically classified into 6 established genogroups (GI-GVI), while tentative genogroup GVII is proposed, and of which GI, GII, and GIV cause diseases in humans [2]. NoV GII.4 has emerged as the predominant genotype causing outbreaks of AGE worldwide. In multivariate analysis, NoV GII.4 were associated with higher hospitalization and mortality rates [3]. In the United States and Europe, NoV are responsible for approximately 50 % of all reported gastroenteritis outbreaks [4]. Similar to rotavirus infection, the main symptoms caused by norovirus infection are diarrhea, vomiting, fever, and even severe dehydration that needs hospitalization. Complications of norovirus AGE in children, such as convulsion, severe dehydration, malnutrition, bowel obstruction and even death were sporadically reported [5, 6]. Significant clinical features correlated with different viral strains has been documented recently, and after implementing rotavirus vaccination, norovirus infection has become one of the most important enteric pathogens threatening human health [3, 7]. Our recent study showed that in Taiwan under suboptimal use of rotavirus vaccines, a significant increase of norovirus infection was observed in the post- rotavirus vaccine era [8].

Disease severity score system for AGE was established previously [9]. The severity score for different clinical features and complications caused by emerging enteric viruses such as NoV is lacking. The purpose of this study was to investigate the clinical features and complications with norovirus infection in hospitalized pediatric patients supposed to have moderate to severe illness, compared to children treated in the community setting, and furthermore, we proposed a disease scoring system modified from Vesakari score system for evaluation of norovirus disease with variable severity in hospitalized patients.

## Methods

### Patient selection and identification of norovial infection

From April of 2004 through December of 2012, pediatric patients with the diagnosis of AGE hospitalized in the Division of Pediatric Gastroenterology, Chang Gung Children’s Hospital (CGCH) were randomly enrolled. Their fecal specimens were collected in a clean container within 3 days of hospitalization with guardian’s informed consent in this study with exclusion of those with major underlying diseases. Norovirus infection was confirmed by the detection of NoV using RT-PCR from fecal specimens of the patients with diarrheal disease [10]. The patient enrollment was irrespective of age, sex, ethnicity, and hospitalization wards. The symptoms were determined and documented in electronic medical records to determine how patient-reported symptoms were addressed by clinicians and clinical data of the patients enrolled were collected retrospectively from the chart records. These studies were approved by the Institutional Review Board of the Chang Gung Memorial Hospital.

### Norovirus genome sequencing and genotyping

Viral nucleic acid extraction from fecal samples was performed using a kit according to the manufacturer’s recommendations (QIAamp Viral RNA Mini Kit; Qiagen, Hilden, Germany). The PCR primers and conditions used for determining NoV genotypes were described previously [10]. The cDNA products were cloned into a plasmid (pCR-XL-TOPO® vector; Invitrogen), and the recombinant plasmid was transferred into competent Escherichia coli (Topo® XL PCR Cloning kit; Invitrogen). The sequences of different PCR products from the same specimen were used to reconstruct the near-full-length norovirus genome using the Vector NTi software package (Invitrogen). The reference genome sequences of NoV used for comparison were all obtained from the National Center for Biotechnology Information database (http://www.ncbi.nlm.nih.gov/).

### Complications of norovirus infection

The complication was recognized and defined as the occurrence of extraintestinal or unusual presentations of viral AGE during hospitalization including abdominal pain or irritability, gastrointestinal (GI) hemorrhage, electrolyte imbalance (hyponatremia: serum sodium level < 135 mmol/L; hypokalemia: serum potassium level < 3.5 mmol/L; or hypochloremia: serum chloride level < 98 mmol/L), prominent hyperthermia (body temperature > 39 °C), convulsive disorder (upward gaze, loss of consciousness, involuntary tonic or clonic manifestations), hypotension or hypovolemic shock (systolic blood pressure < 70 mmHg), hypoglycemia (serum sugar level < 70 mg/dL).

### Modified score system for severity of norovirus infection

We evaluated the disease severity of norovirus infection by the Vesikari score system (total score < 7, mild; 7-≦10, moderate, and >10, severe. total maximum score 20) [9] and a modified score system based on clinical information from the hospitalized patients (Table 1). The modified severity score system has a 0–24 point numerical score, including the assessment for the presence of unusual clinical features, such as gastrointestinal hemorrhage (gross bloody stool or occult blood ≧ 2+ in stool), convulsion, and additional presentations such as abdominal pain or flatulence and prolonged or high fever. The modified system has a more simple stratification of general AGE symptoms from 3 points to 2 points hence with a total maximum score of 24. In our 24 points score system, mild degree is as less than 1/3 maximum score (8) and severe degree is half score or higher (12), that is score, < 8, mild; 8-≦11, moderate, and >11 (12 or higher) severe. The differences of the two score systems are listed in Table 1.

**Table 1:** Comparison of Vesikari and modified Vesikari score systems for acute gastroenteritis

Symptom or sign	Vesikari Score System (Points)	Modified Score System (Points)
Max. no. diarrheal stools/24 h		
1–3	1	1
4–5	2	2^a^
≥ 6	3	
Duration of diarrhea (days)		
1–4	1	1
5	2	2^a^
≥ 6	3	
Max. no. vomiting episodes/24 h		
1	1	1^a^
2–4	2	
≥ 5	3	2
Duration of vomiting (days)		
1	1	1^a^
2	2	
≥ 3	3	2
Fever		
< 37.0 °C	0	0
37.1–38.4 °C	1	1
38.5–38.9 °C	2	2
≥ 39 °C	3	3
Dehydration		
Little to mild	1	1
Mild to Moderate (1–5 %)	2	2
Severe (≥6 %)	3	3
Treatment		
None	0	0
Rehydration	1	1^b^
Hospitalization	2	2^c^
Duration of fever (days)		
0	N/A	0
1–3	N/A	1
≥ 3	N/A	2
Gastrointestinal hemorrhage		
None	N/A	0
Occult blood in stool only	N/A	1^d^
Gross bloody stool	N/A	2
Convulsion		
None	N/A	0
Yes	N/A	1
With recurrence		2
Assessment of abdomen pain or flatulence		
None	N/A	0
Flatulence	N/A	1
Irritability or pain	N/A	2
Total Score	20	24

Total score in Vesikari score < 7, mild; 7-≦10, moderate, and >10, severe. In modified score, < 8, mild; 8-≦11, moderate, and >11, severe

*N/A* Not assessed

^a^ Max. no. diarrheal stools/24 h ≥ 4; Duration of diarrhea ≥ 5 days; Max. no. vomiting episodes/24 h ≦ 4; Duration of vomiting ≦ 2 days

^b^ Hydration amount of less than 1.5 fold maintenance

^c^ Hydration amount of more than 1.5 fold maintenance

^d^ Occult blood (≥) 2+ in stool

### Statistical analysis

The chi-squared test or Fisher’s exact test was applied to dichotomous variable analysis, and the unpaired-sample student’s *t*-test was used for continuous variable analysis. A p value of < 0.05 was considered statistically significant. All the tests were analyzed using SAS system software version 8 for Windows.

## Results

### Outbreaks of norovirus acute gastroenteritis

During the period from 2004 to 2012, there were 957 hospitalized pediatric patients fulfilled study criteria initially enrolled. After the exclusion of 11 with bacterial infection, 7 finally diagnosed as specific disease other than infections, and 19 with incomplete clinical data collection, a total of 920 pediatric patients were finally enrolled. Among them, 207 (22.5 %) were positive for NoV in their fecal samples by RT-PCR method and 189 after excluding 18 with mixed infections, including 10 with rotavirus, 3 with astrovirus, 2 with enteric adenovirus, 1 with sapovirus, 1 with rotavirus and astrovirus, and 1 with *Salmonella*. The age distridution of the 920 enrolled children was 18 day- 247 months old (Mean: 31.6 months, median: 24 months), and of norovirus infection was 1–240 months old (mean 26.7 months, median: 17 months). Norovirus infection was confirmed in patients admitted in the four major outbreaks of 2004/2005 winter, 2006/2007 winter, 2008/09/10 winter, and 2011/2012 winter in 35 (11.6 %), 75 (41.4 %), 36 (15.9 %), and 43 (20.5 %) patients, respectively.

### Complications of norovirus infection in different time periods

**Fig. 1:**
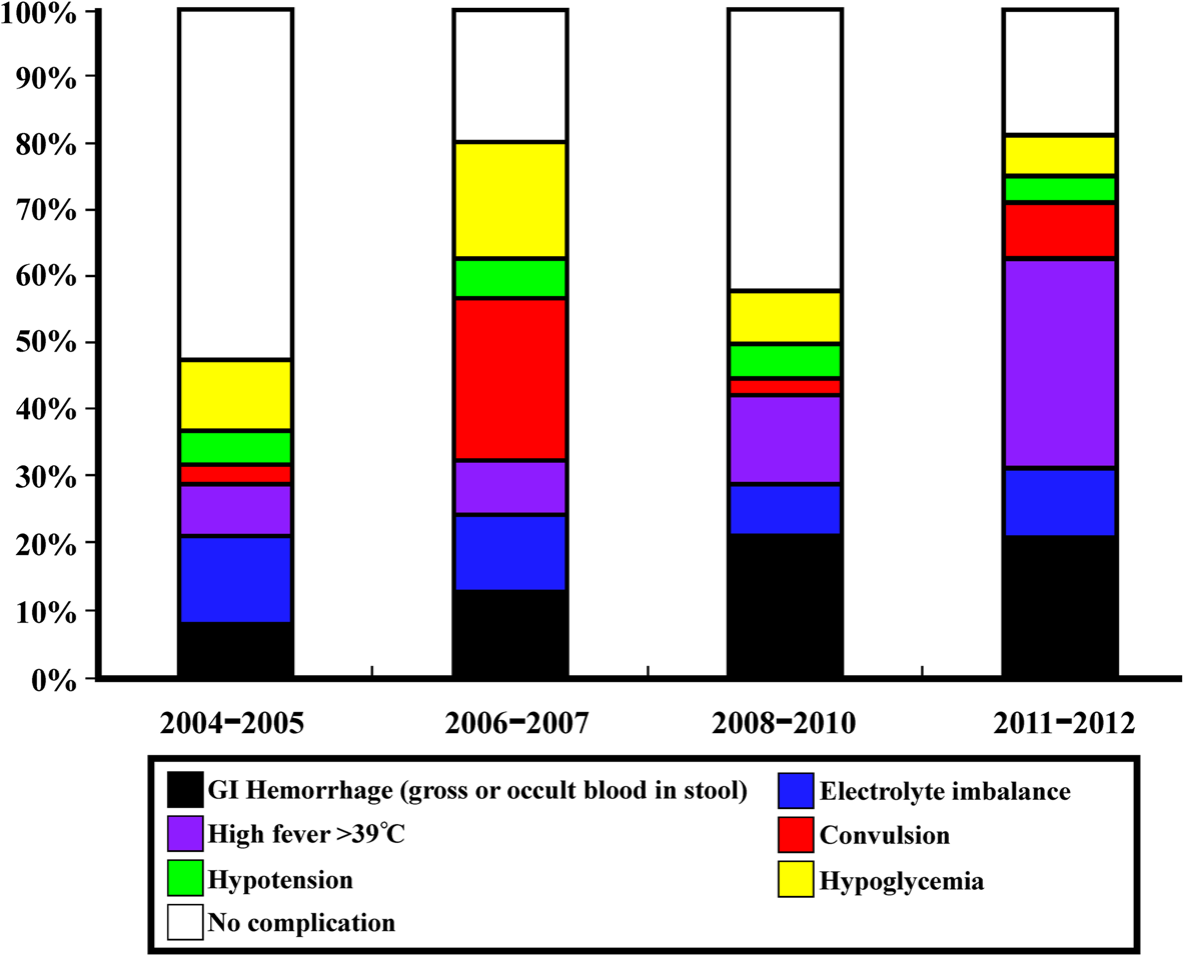
Complications in different periods of norovirus outbreaks in northern Taiwan. The total numbers of hospitalized patients with confirmed norovirus infection during each outbreak are 35 in 2004–2005, 75 in 2006/2007 winter, 36 in 2008/09/10 winter, and 101 in 2011/12 winter. The most common complications are electrolyte imbalance (14.3 %) in 2004/2005 winter, convulsion (28 %) 2006/2007 winter, gastrointestinal (GI) hemorrhage (22.2 %) 2008/09/10 winter, and high fever (34.9 %) 2011/12 winter

Figure 1 and Table 2 demonstrate the complications of norovirus infection observed in different time periods. From 2004 to 2005 winter, the most common complications were electrolyte imbalance (14.3 %) and hypoglycemia (11.4 %). From 2006 to 2007 winter, the most common complications were 28 % for convulsive disorder and 20 % for hypoglycemia. In 2008/09/10 winter, major complications including GI hemorrhage (22.2 %) and prominent hyperthermia (13.8 %) were observed. In the latest period from 2011 to 2012 winter, norovirus infection caused prominent hyperthermia (34.9 %) and GI hemorrhage (23.3 %) of the patients that were the most common complications.

**Table 2:** Complications and uncommon clinical manifestations of norovirus infection in 2004–2012 in northern Taiwan

% Symptoms	2004/2005 winter (N = 35)	2006/2007 winter (N = 75)	*P* -value ^*b*^	2008/09/10 winter (N = 36)	*P*-value	2011/12 winter (N = 43)	*P*-value
Abdominal pain or irritability	22.9 (8)	21.3 (16)	0.937	41.7 (15)	0.033	44.2 (19)	0.014
GI Hemorrhage (gross and occult blood in stool)	8.6 (3)	14.7 (11)	0.217	22.2 (8)	0.047	23.3 (10)	0.030
Electrolyte imbalance	14.3 (5)	13.3 (10)	0.984	8.5 (3)	0.475	11.6 (5)	0.816
Fever >38 °C	37.1 (13)	36 (27)	0.814	25 (9)	0.208	62.8 (27)	0.015
High fever >39 °C	8.6 (3)	9.3 (7)	0.722	13.8 (5)	0.330	34.9 (15)	0.001
Convulsion	2.9 (1)	28 (21)	<0.001	2.8 (1)	0.798	9.3 (4)	0.266
Hypotension	5.7 (2)	6.7 (5)	0.817	5.5 (2)	0.983	4.6 (2)	0.825
Hypoglycemia	11.4 (4)	20 (15)	0.316	8.5 (3)	0.475	6.9 (3)	0.320
Disease severity score > 10 (severe)	22.9 (8)	26.6 (20)	0.604	30.6 (11)	0.403	23.3 (10)	0.943
Modified severity score > 11 (severe)	28.5 (10)	32 (24)	0.643	33.3 (12)	0.572	30.2 (13)	0.748
Predominant genotype/subgenotype, No. (%)^*a*^	ND	GII.4 2006b, (Den_Haag) 28 of 70 (40 %)		GII.4 2010, (New Orleans) 16 of 33 (48.5 %)		GII.4 Sydney, 11 of 37 (29.7 %) GII.4 2012b, 10 of 37 (27 %)	

^a^ Percentage of dominant strains in samples available for sub-genotype analysis

^b^ P-value means statistical results of compariing each item in later year period to those in the earlier years of 2004–2005

Compared to norovirus infection in 2004/2005 winter, significant higher incidence of complications in the later periods are: in 2006/2007 winter, convulsive disorder (*p* < 0.001); in 2008/09/10 winter, GI hemorrhage (*p =* 0.047) and severe abdominal pain or irritability (*p =* 0.033); in 2011/2012 winter, GI hemorrhage (*p* = 0.030), severe abdominal pain or irritability (*p =* 0.014), and prominent hyperthermia (fever >39 °C) (*p =* 0.001).

### Circulating NoV strains and complications

According to the genomic analysis of NoV, GII.4 was the most common genotype in all time periods; the major circulating NoV subgenotype or variant in 2006/2007 winter, GII.4 Den_Haag_2006b (28 of 70, 40 %), caused most complications of convulsion (19 of 28, 67.9 %) in infected children. The major subgenotype in 2008/09/10 winter, GII.4 2010 (New Orleans) (16 in 33, 48.5 %), caused GI hemorrhage (6, 37.5 %). Furthermore, GII.4 2012 subgenotypes (21 in 37, 56.8 %), the predominat NoV strains in 2011/2012 winter, caused more high fever (12, 57.1 %) and GI hemorrhage (7, 33.3 %).

### Disease severity assessment by a modified score system compared to Vesikari score system

With regards to assessment using Vesikari score system, the overall distribution of patients’ disease severity was mild (score < 7) in 58 patients (30.6 %), moderate (7 ≦ score ≦ 10) 83 (43.9 %), and severe (score > 10) 49 (25.9 %). The proportions of patients with severe disease were 22.9 %, 26.6 %, 30.6 %, and 23.3 % in the four periods, and it was usually lower than 30 % (in 3 of the 4 periods). Hospital stays related to disease severity (median durations with interquartile ranges) are shown in Table 3: mild level, 5 (4–5) days; moderate, 4 (3–6) days; and severe, 4 (4–5) days, with the longest hospitalization in mild level group (Table 3). Under the assessment by modified score method (Table 1), the distribution of patients’ disease severity is mild (score < 8) in 50 (26.5 %), moderate (8 ≦ score ≦ 11) in 80 (42.3 %), and severe (score > 11) in 59 (31.2 %). The proportions of severe status are 28.5 %, 32 %, 33.3 %, and 30.2 % with the highest found in 2008/09/10 winter. Hospital stay according to severity assessed by modified score system (median durations with interquartile ranges) were: mild disease, 3 (3–4) days; moderate, 5 (4–6) days; and severe, 5 (4–7) days, with the longest hospitalization in severe disease group (*p* = 0.002) (Table 3). A longer duration of hospitalization (*p* = 0.02) in those with a high score under modified Vesikari score system rather than by the original Vesikari score system was observed.

**Table 3:** Stratification of patients’ disease severity by different score systems

Characteristics	Vesikari Score System	Modified Vesikari Score System	*P*-value ^a^
Proportion No (%)			
Mild	58(30.6 %)	50 (26.5 %)	0.437
Moderate	82 (43.9 %)	80 (42.3 %)	0.582
Severe	49 (25.9 %)	59 (31.2 %)	0.283
Hospitalization (days)			
Mild	5 (4 ~ 5)^b^	3 (3 ~ 4)	0.01
Moderate	4 (3 ~ 6)	5 (4 ~ 6)	0.28
Severe	4 (4 ~ 5)	5 (4 ~ 7)	0.027

^a^ Statistical comparison of disease severity assessed by different score systems

^b^ The mean (interquartile range, 25 % ~ 75 %) of hospitalization days

## Discussion

NoV is one of the emerging pathogens causing enteric infections in humans, and the associated clinical presentations are diverse [11–14]. Other than gastrointestinal symptoms, norovirus infection is associated with several extraintestinal or unusual complications, including benign infantile convulsion [5], necrotizing enterocolitis [15], and exacerbations of inflammatory bowel disease [16], as well as chronic, serious outcomes in immunocompromised patients [17, 18]. Comprehensive genotyping methods have been applied to monitor the molecular epidemiology of norovirus infections worldwide. Association of a given genotype or subgenotype with the occurrence of complications following gastroenteritis has been reported: GII.4 Den_Haag_2006b subtype caused infantile seizure following gastroenteritis during 2006–2007 outbreaks in Taiwan and GII.3 infection caused necrotizing enterocolitis and neonatal death in a neonatal intensive-care unit in 1998 in the United States [5, 15]. Although the specific NoV subgenotypes related to severe infection and complications was only sporadically reported, the rapid genetic evolution of NoV is believed to be the main factor driving the changing clinical manifestations in the infected patients. On the other hand, there is an unmet need that we need an objective disease severity score system specifically for the assessment of the severity of norovirus infection, as the Vesikari score used in rotavirus vaccine trials [19, 20]. Recently, a modified Vesikari score system for severity assessment of AGE in children in the emergency departments has been devised [21]. This is a simplified version of Vesikari score system by the deletion of dehydration status as a score item. Nevertheless, the evidence-based guidelines in Europe highlighted that enteric infectious agent is frequently associated with dehydration which reflects severity of disease and should be monitored by established severity score systems [22]. Complications of AGE and dehydration usually require aggressive management in norovirus infection. Thus, the addition of clinical complications as score items to form a modified system for severity assessment of norovirus infection is necessary. Based on this, we constructed such a modified system and verified its performance in assessing norovirus infection in different outbreaks in this study. A longer hospitalization in patients classified into mild severity in Veskari score system could be due to a prolonged course of fever, and presence of complications, abdominal pain and flatulence not included in Vesikari score system. These symptoms may cause poor appetite and delayed recovery of activity that require intravenous hydration. Furthermore, a significantly longer hospital stay in the higher-scored patients was found as practically expected because it is complication-weighted. Thus, the newly devised modified score system could be a useful tool in clinical practice.

There are several limitations in our study. First of all, our study enrolled hospitalized children usually having a moderate or severe illness rather than children with mild illness treated in a community setting. Secondly, with regards to the NoV subgenotypes, we could only verify the major strains circulating in each outbreak. Thirdly, although all the fecal samples were tested for bacterial pathogens and viruses, and NoV was found the only agent positive in these specimens, still other pathogens that may be associated with the clinical manifestations are not included in the panel. Fourthly, in spite of the presence of the complications in norovirus AGE, the mechanisms for the occurrence of such intestinal or extra-intestinal complications remain to be determined.

## Conclusions

Vaccine development for the prevention of norovirus infection is currently under way and a more objective assessment of the infection is of paramount importance [23–27]. This study demonstrated that different circulating NoV subgenotypes were associated with different complications and uncommon presentations in a series of outbreaks over time in northern Taiwan. The stratification of hospitalized patients by assessment of the severity of their disease severity using a modified severity score system is possible, suggesting that the system is useful for future outbreak investigation. NoV is a rapidly evolving virus with the circulating genotypes or subgenotypes varying over time.

## Abbreviations

AGE: Acute gastroenteritis
CGCH: Chang Gung Children’s Hospital
GI: Gastrointestinal
NoV: Norovirus
